# Surgical Injury and Ischemia Prime the Adipose Stromal Vascular Fraction and Increase Angiogenic Capacity in a Mouse Limb Ischemia Model

**DOI:** 10.1155/2020/7219149

**Published:** 2020-05-18

**Authors:** Satoko Kishimoto, Ken-ichi Inoue, Ryoichi Sohma, Shigeru Toyoda, Masashi Sakuma, Teruo Inoue, Ken-ichiro Yoshida

**Affiliations:** ^1^Comprehensive Research Facilities for Advanced Medical Science, Research Center for Advanced Medical Science, Dokkyo Medical University, Mibu, Tochigi 321-0293, Japan; ^2^Center of Regenerative Medicine, Dokkyo Medical University Hospital, Mibu, Tochigi 321-0293, Japan; ^3^Department of Cardiovascular Medicine, Dokkyo Medical University, Mibu, Tochigi 321-0293, Japan

## Abstract

The adipose-derived stromal vascular fraction (SVF) is an effective source for autologous cell transplantation. However, the quality and quantity of SVFs vary depending on the patient's age, complications, and other factors. In this study, we developed a method to reproducibly increase the cell number and improve the quality of adipose-derived SVFs by surgical procedures, which we term “wound repair priming.” Subcutaneous fat from the inguinal region of BALB/c mice was surgically processed (primed) by mincing adipose parenchyma (injury) and ligating the subcutaneous fat-feeding artery (ischemia). SVFs were isolated on day 0, 1, 3, 5, or 7 after the priming procedures. Gene expression levels of the primed SVFs were measured via microarray and pathway analyses which were performed for differentially expressed genes. Changes in cellular compositions of primed SVFs were analyzed by flow cytometry. SVFs were transplanted into syngeneic ischemic hindlimbs to measure their angiogenic and regeneration potential. Hindlimb blood flow was measured using a laser Doppler blood perfusion imager, and capillary density was quantified by CD31 staining of ischemic tissues. Stabilization of HIF-1 alpha and VEGF-A synthesis in the SVFs were measured by fluorescent immunostaining and Western blotting, respectively. As a result, the number of SVFs per fat weight was increased significantly on day 7 after priming. Among the differentially expressed genes were innate immunity-related signals on both days 1 and 3 after priming. In primed SVFs, the CD45-positive blood mononuclear cell fraction decreased, and the CD31-CD45-double negative mesenchymal cell fraction increased on day 7. The F4/80-positive macrophage fraction was increased on days 1 and 7 after priming. There was a serial decrease in the mesenchymal-gated CD34-positive adipose progenitor fraction and mesenchymal-gated CD140A-positive/CD9-positive preadipocyte fraction on days 1 and 3. Transplantation of primed SVFs resulted in increased capillary density and augmented blood flow, improving regeneration of the ischemic limbs. HIF-1 alpha was stabilized in the primed cutaneous fat *in situ*, and VEGF-A synthesis of the primed SVFs was on a peak on 5 days after priming. Wound repair priming thus resulted in SVFs with increased number and augmented angiogenic potential.

## 1. Introduction

The adipose-derived stromal vascular fraction (SVF) is a cell population derived from enzymatic digestion of adipose tissue [1]. Freshly isolated SVFs not only are a rich source of adipose-derived stem/stromal cells but also contain a heterogeneous mixture of cells, including endothelial progenitor cells, endothelial cells, smooth muscle cells, pericytes, fibroblasts, mesenchymal cells, lymphocytes, macrophages, and preadipocytes [2]. Autologous transplantation of SVFs is a fast-growing angiogenesis therapy. The advantages of this protocol include an abundant supply of cells, ease of isolation without cell culture, and no risk of transplant rejection. However, it is practically difficult to ensure uniform SVF quality in clinical settings. Several reports describe that ageing [3–5] and diabetes [6] may impair the quality and angiogenic functional capacity of SVFs. To achieve reproducibility and effectiveness with SVF transplantation, it is necessary to overcome problems with individual variation in SVF quality.

Here, we show that surgical treatments for adipose tissue reproducibly increase and reorganize adipose-derived SVFs. We term this phenomenon “wound repair priming” and developed a novel method to reproducibly obtain SVFs with increased number and good quality. The primed SVFs not only have increased cell numbers but also have enhanced angiogenesis capacity during the regeneration of ischemic limbs. Both injury-associated stimuli and ischemia are needed for the priming, and immune cells are heavily involved through heterogeneous cell-cell communications. We investigated the boosted performance of primed SVFs over nonprimed SVFs to promote angiogenesis and regeneration in a mouse limb ischemia model.

## 2. Materials and Methods

### 2.1. Preliminary Discovery and Reproducing the Phenomenon of “Wound Repair Priming”

All animal experiments adhered to the Guidelines for Animal Experimentation of Dokkyo Medical University, with all effort taken to minimize animal numbers and suffering. Inbred male BALB/c mice (CLEA Japan, Inc., Shizuoka, Japan) aged 9–12 weeks were anesthetized with an intraperitoneal injection of 90 mg/kg ketamine hydrochloride (Ketalar; Daiichi Sankyo Propharma Co., Ltd., Tokyo, Japan) and 10 mg/kg xylazine hydrochloride (Celactal; Bayer Yakuhin, Ltd., Osaka, Japan). SVFs were isolated from subcutaneous inguinal fat in BALB/c mice and transplanted into a syngeneic limb ischemia model as described previously [7].

In a preliminary experiment, we observed that the number of SVFs was dramatically increased when a mouse received skin injury by fighting with a littermate (data not shown). When we transplanted the SVFs (adjusted to the same number) of a donor mouse receiving a skin injury to the ischemic limbs of a syngeneic mouse, the capacity of angiogenesis was greatly enhanced (data not shown). We found that the transplantation of SVFs from an injured mouse displayed much faster blood flow recovery than that from an uninjured mouse. Therefore, we hypothesized that adipose tissue injury substantially increases the number and quality of SVFs.

We then designed the present study to reproduce this phenomenon in a controlled condition and to investigate the underlying mechanisms. A few days before isolating SVFs, adipose tissue was surgically damaged. The priming models were classified into 3 groups based on the following procedures: mincing fat parenchyma (injury), ligating the subcutaneous fat-feeding artery (ischemia), and both (injury+ischemia group). A sham operation group, which underwent cutting and suturing of the epidermis but with no surgical damage to fat tissues, was also used.

### 2.2. Preparation of SVFs

SVFs were isolated from inbred BALB/c mice with wound repair priming (injury only, ischemia only, and injury+ischemia) or sham operation (nonprimed SVFs) as previously described [8, 9], with several modifications. BALB/c mouse adipose tissue from the inguinal region was removed, minced, transferred to C tubes (Miltenyi Biotec Corp., Tokyo, Japan), and digested with 0.1% collagenase type I (Wako Pure Chemical Industries, Ltd., Osaka, Japan) and 0.2% dispase type II (Life Technologies) for 1 h at 37°C. The digested tissue was mechanically and gently dispersed with a MACS Dissociator (Miltenyi Biotec Corp., installed software program “m_brain01-02”) every 10 min. The suspension was passed through a 100 *μ*m filter (BD Falcon, Franklin Lakes, NJ), centrifuged at 420 *g* for 5 min (LC-200; Tomy Seiko Co., Ltd., Tokyo, Japan), and resuspended in Dulbecco's Modified Eagle's Medium (DMEM; Life Technologies Oriental, Tokyo, Japan). The number of cells stained with 0.4% Trypan blue and counted using a hemocytometer.

### 2.3. Microarray and Pathway Analyses

Total RNA was purified from SVFs after the priming procedure using phenol-chloroform extraction (RNAiso Plus, Takara Bio Inc., Shiga, Japan). For comparison, three time points were taken (0, 1, or 3 days after). Three individual mice were used for each time point for statistical analysis. After confirming the quality of RNA (2100 Bioanalyzer, Agilent Technologies, Santa Clara, CA, USA), gene expression levels were measured by microarray analysis (Affymetrix GeneChip Expression Array, Mouse430_2, Thermo Fisher Scientific, Inc., Waltham, MA, USA). Differentially expressed genes were selected using Affymetrix Transcriptome Analysis Console (TAC, Ver3.1.0.5) software. Filtering criteria were as follows: (1) the expression ratio was less than half or more than double, and (2) the *p* value of repeated-measurements analysis of variance was less than 0.05. The list of differentially expressed genes was subsequently imported to Ingenuity Pathway Analysis software (Qiagen, Hilden, Germany). Fisher's exact test was used to estimate the “enrichment” of differentially expressed genes among each pathway or functional ontology. In canonical pathway analysis, the activation *z*-score was calculated based on the curated information about “seed molecules” and the alterations of the seeds among the dataset. In the upstream and downstream (diseases and functions) analyses, activation *z*-scores were calculated based on the consistency between knowledge-based effects (ingenuity knowledge base) and the patterns observed among the dataset.

### 2.4. Western Blotting

SVFs with or without priming were isolated, and whole-cell proteins were denatured with a solubilizer (7 M urea, 2 M thiourea, and 4% CHAPS, Thermo Fisher). Protein concentrations were measured using the Bradford protein assay kit (Bio-Rad Laboratories, Inc., Hercules, CA, USA) and plate reader (Infinite F200 PRO, Tecan, Zurich, Switzerland). Proteins were subsequently diluted in lithium dodecyl sulfate (NuPAGE Sample Buffer, Thermo Fisher Scientific, Inc., Waltham, MA, USA) at the concentration of 2 mg/mL and reduced in 50 mM dithiothreitol. The entangled genomic DNA was sheared by pulsed sonication for 30 min at 4°C (Bioruptor® II, Sonic Bio Inc., Kanagawa, Japan). Proteins were size-fractionated by electrophoresis in 12% sodium dodecyl sulfate polyacrylamide gel, then wet-transferred to a PVDF membrane (Immobilon-P, Merck Millipore, Billerica, MA, USA). After blocking with 2% skim milk TBST (Tris/HCl pH 7.4, 150 mM NaCl, and 0.1% Tween 20), the membranes were incubated with VEGF-A antibody (#AB1876-I, Merck Millipore) or GAPDH antibody (#5174, Cell Signaling Technology, Danvers, MA, USA) at 4°C overnight. Nonspecific binding was washed away with TBST, and the protein-antibody complex was visualized using horseradish peroxidase-conjugated secondary antibody (#7074, Cell Signaling Technology) and the luminescence reaction (#WSE-7120 or 7110, Atto, Tokyo, Japan). Images were taken with a CCD camera (LuminoGraph I, Atto).

### 2.5. Flow Cytometry

Subcutaneous fat prepared from the inguinal region of the mice was sampled on days 1, 3, and 7 after priming (injury+ischemia). These samples were minced into small pieces and digested with collagenase in phosphate-buffered saline (PBS) with Ca^2+^ and Mg^2+^ containing 2% bovine serum albumin (BSA) at 37°C for 45 min using a gentleMACS Octo Dissociator with Heaters (Miltenyi Biotec GmbH, Bergisch Gladbach, Germany). The samples were passed through a 100 *μ*m nylon mesh and subsequently treated with DNase I (Roche Diagnostics GmbH, Mannheim, Germany). Cells were washed with PBS with 0.5% BSA and 2 mM ethylenediaminetetraacetic acid (EDTA) (FACS buffer), hemolyzed, and resuspended in FACS buffer for downstream experiments.

Flow cytometry was used to determine cell surface marker presentations of SVFs [1, 10, 11]. Briefly, the cells were incubated with anti-mouse Fc*γ*RII/III (2.4G2, BD Biosciences, San Jose, CA) for 10 min at 4°C and then stained with the following anti-mouse antibodies: FITC-conjugated antibodies specific for CD34, PE-conjugated anti-CD31, PerCP-Cy5.5-conjugated anti-CD45, APC-conjugated anti-CD140A (PDGFR alpha; BD Biosciences), and FITC-conjugated anti-CD9 (Thermo Fisher Scientific, San Diego, CA). To identify M1 or M2 macrophages, we used FITC-conjugated anti-F4/80, PE-conjugated anti-CD11c, and Alexa Fluor 647-conjugated anti-CD206 (AbD Serotec, Oxford, UK). The M1 and M2 macrophages were identified as F4/80-positive/CD11c-positive/CD206-negative and F4/80-positive/CD11c-negative/CD206-positive cells, respectively. Appropriate nonspecific isotypes were used as controls. Cells were analyzed with a FACSCalibur flow cytometer (Becton Dickinson, Mountain View, CA) using CellQuest software (Becton Dickinson).

### 2.6. Mouse Hindlimb Ischemia Model and Experimental Protocol

Mice underwent ligation of the right external iliac artery and hindlimb vein to produce right hindlimb ischemia [12]. Mice were randomly allocated into five groups (*n* = 6 each): control group (no operation), sham operation group (no SVF injection), injury-only group, ischemia-only group, and injury+ischemia group. SVFs were injected at eight different sites (5 × 10^5^ cells; 20 *μ*L per site) on the adductor muscles of the ischemic limb one day after the surgery. This 24-hour interval between the artery ligation and SVFs' injection makes the model so severe that the nonprimed SVFs displayed no effect, resulting in the hindlimb falling off.

### 2.7. Hindlimb Blood Flow Assessment

Hindlimb blood flow was analyzed using a laser Doppler blood perfusion imager (PeriScan PIM III; Perimed AB, Stockholm, Sweden) on postoperative day 0 (within 1 h of the operation) and on days 3, 7, and 14. A depilatory cream was used to remove excess limb hair before imaging. Mice were placed on a heating plate at 38°C to minimize temperature variation during imaging. Blood perfusion was calculated on the scanned images as perfusion units (PU), and serial changes in the ischemic (right) hindlimb were compared among the groups. Changes in blood perfusion for the contralateral nonischemic (left) hindlimb blood perfusion were also assessed to avoid variation bias resulting from ambient light and temperature.

### 2.8. Immunohistochemistry

Paraffin-embedded sections (5 *μ*m thick) underwent standard deparaffinization and rehydration procedures. The sections were treated with antigen retrieval solution (pH 9) (Agilent Technologies, Santa Clara, CA, USA) for 1 min at 105°C by autoclaving. The sections were then blocked with 3% (v/v) normal bovine serum albumin (BSA) in phosphate-buffered saline (PBS) for 20 min. Ischemic thigh adductor skeletal muscle tissue samples were obtained on days 7 and 14 after injection. The presence of collateral vessels that bypassed the occluded segment of the femoral artery was assessed by immunohistochemical staining with rat anti-mouse CD31 (Dianova, Hamburg, Germany) and evaluation of five random microscopic fields per slide by light microscopy (BZ-X710; Keyence Corp., Osaka, Japan). Staining was performed using the avidin-biotin complex method (Vectastain Elite ABC kit; Vector, Burlingame, CA) with diaminobenzidine (DAB substrate kit; Zymed, S. San Francisco, CA) as the peroxidase substrate. The CD31-positive vessel area was quantified as the percentage of the total tissue area in a high-power field (400x) using Adobe Photoshop CS6 (Adobe Systems Inc., San Jose, CA). A threshold was selected and used to identify positive pixels on each slide. The percent area was calculated as the percentage of positive pixels relative to the total number of pixels in each view. Adjacent tissue sections were stained with hematoxylin and eosin [13]. Cutaneous fat tissue samples were obtained on day 1 after priming. The samples were incubated with rabbit anti-mouse hypoxia-induced factor- (HIF-) 1 alpha (#36169, Cell Signaling Technology) overnight at 4°C. The samples were then incubated with the secondary antibodies Alexa Fluor 594 goat anti-rabbit IgG (#8889, Cell Signaling Technology) for 1 h at room temperature. After the sections were counterstained and mounted with ProLong Gold Antifade with DAPI (Cell Signaling Technology). Microscopic observation and photography were performed using a fluorescence microscopy (BZ-X710).

### 2.9. Statistical Analysis

Data are shown as mean ± standard deviation (SD). The Mann-Whitney *U* test was used for two-group comparisons. Data for flow cytometry and cell counts were analyzed using nonparametric Fisher's LSD method: Kruskal-Wallis one-way analysis of variance followed by the Mann-Whitney *U* test as a *post hoc* analysis. *p* values < 0.05 were considered significant. Statistical analysis was performed using SPSS version 25 (IBM Corp., Armonk, NY, USA).

## 3. Results

### 3.1. Surgical Injury and Ischemia (Priming) Increased the Live Cell Number of SVFs

**Figure 1:**
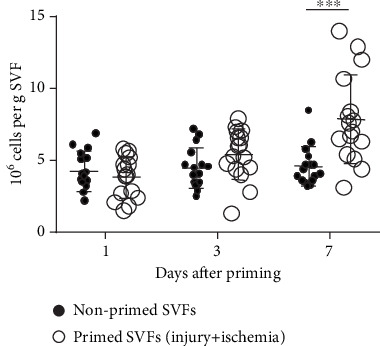
Surgical injury increased the live cell number of SVFs. The number of SVFs per tissue weight. Primed SVFs were created by both mincing fat parenchyma (injury) and ligating the subcutaneous fat-feeding artery (injury+ischemia). The error bars represent the standard deviation of measurements from 16 samples. ^∗∗∗^*p* < 0.001 primed vs. nonprimed SVFs. SVF: stromal vascular fraction.

We counted the cell number of surgically treated (injury+ischemia) SVFs to evaluate the impact of wound repair priming. The live cell number of primed SVFs increased on day 7 compared with the baseline, whereas the live cell number of nonprimed SVFs did not change on day 1, 3, or 7. The total live cell number of primed SVFs was 1.72-fold higher (*p* < 0.001) on day 7 compared with that of nonprimed SVFs (Figure 1). The survival rate of primed SVFs was 86.9 ± 9.94% (range, 59.1–100%) on day 1, 89.81 ± 9.78% (range, 67.8–100%) on day 3, and 91.1 ± 5.43% (range, 77–97.3%) on day 7. The survival rate of nonprimed SVFs was 82.76 ± 7.47% (range, 66.7–91.7%) on day 1, 86.14 ± 8.79% (range, 71–98.3%) on day 3, and 77.33 ± 8.60% (range, 59.2–94.7%) on day 7. There was no significant difference of the survival rate between the primed and nonprimed SVFs.

### 3.2. Priming (Injury+Ischemia) Dramatically Altered the Gene Expression Profile of SVFs

**Figure 2:**
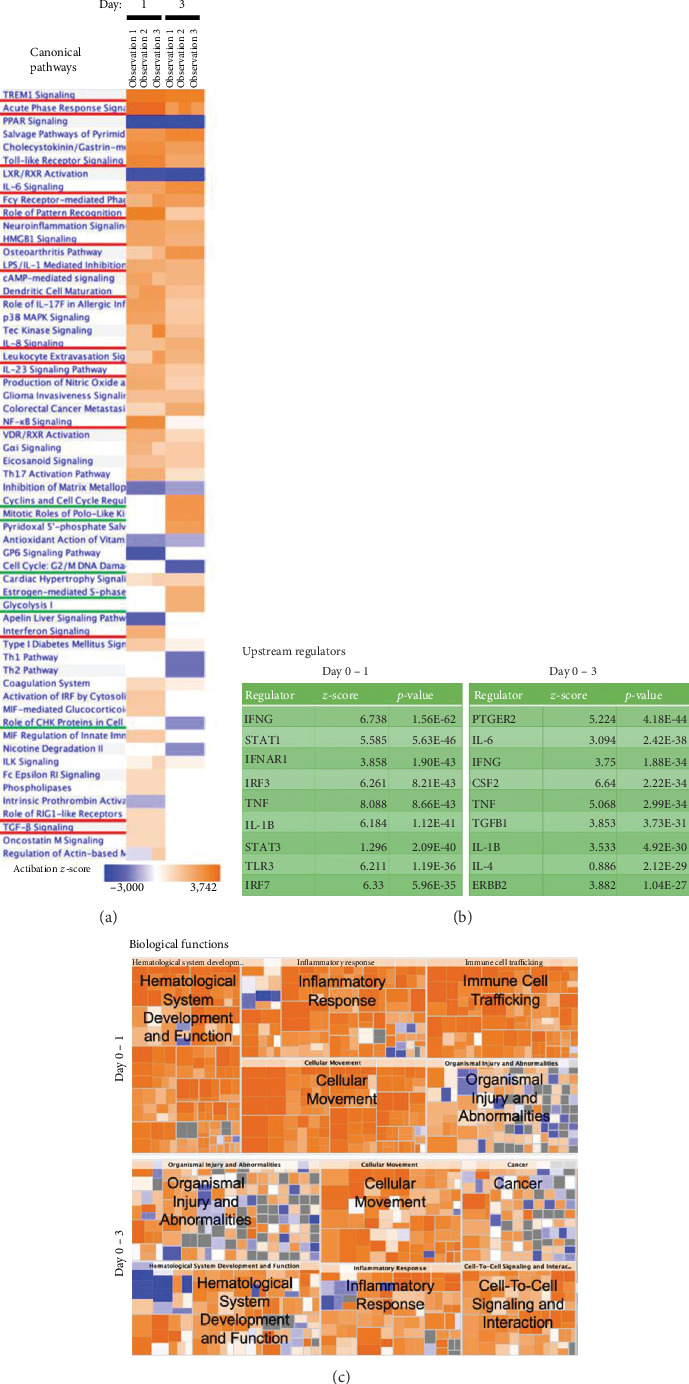
Canonical pathways and upstream-downstream effects involved in SVF priming. Gene expression profiles were compared between primed SVFs and nonprimed SVFs (i.e., 0 vs. 1 day or 0 vs. 3 days after priming), and the lists of differentially expressed genes were imported into Ingenuity Pathway Analysis software. (a) Canonical pathways were clustered according to the activation *z*-score. The heat map indicates the *z*-score based on alterations of “seed molecules” among the dataset. Each column indicates a repeat of experiments (observation) from a distinct time point (comparisons of 0 vs. 1 day and 0 vs. 3 days). The clustering overrepresented immune-related pathways, especially those of innate immunity (red underlines). Cell cycle-related pathways were overrepresented on day 3 after the priming (green underlines). (b) Lists of upstream regulators, the *p* values (Fisher's exact test) of which were the lowest. (c) Expected biological functions downstream of the gene expression changes are shown. Tiled boxes represent the functionally curated gene groups, the area of which is inversely correlated with the *p* value (Fisher's exact test). Small boxes are assembled in a larger box, creating a more common biological annotation. The color of each box indicates the activation *z*-score, although the algorithm is different from that of canonical pathway analysis. Grey-shaded boxes indicate that it was infeasible to determine activation or inactivation. SVF: stromal vascular fraction.

**Figure 3:**
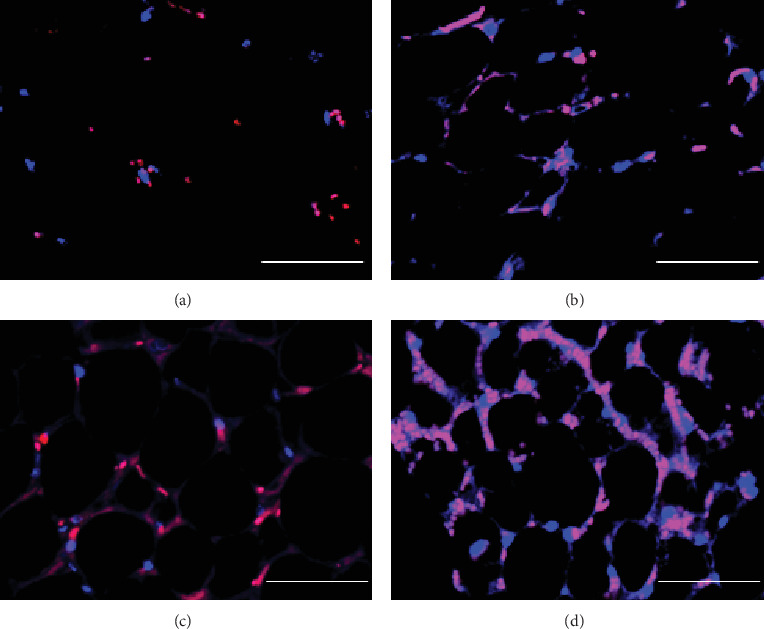
Stabilization of HIF-1 alpha with or without surgical treatments in cutaneous fat *in situ*. Representative images of HIF-1 alpha stabilization *in situ* on day 1 after the surgical treatments. (a) Sham operation group. (b) Injury-only group. (c) Ischemia-only group. (d) Injury+ischemia group. The remarkable stabilization of HIF-1 alpha was observed in the injury+ischemia group. However, sufficient stabilization of HIF-1 alpha was not observed in the injury-only group and the ischemia-only group. Scale bars = 50 *μ*m. HIF: hypoxia-induced factor.

**Figure 4:**
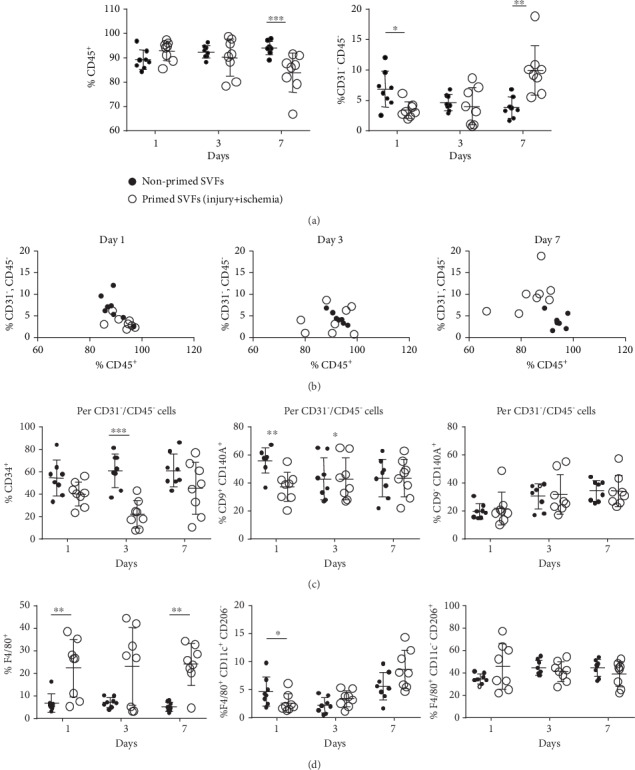
Change in cellular compositions of SVFs by priming. Flow cytometry analysis of surface antigens in primed (injury+ischemia) and nonprimed SVFs. (a) CD45-positive mononuclear (left) and CD31-CD45-double negative (right) mesenchymal cell fractions on days 1, 3, and 7 after priming. (b) Scatterplots of CD45-positive and CD31-CD45-double negative cells are shown. Note that the primed and nonprimed SVFs were clearly segregated on day 7 after priming. (c) Mesenchymal-gated CD34-positive adipose progenitors, mesenchymal-gated CD140A-positive/CD9-high preadipocytes, or mesenchymal-gated CD140A-positive/CD9-low prefibroblasts on days 1, 3, and 7 after priming. (d) F4/80-positive macrophages, F4/80-positive/CD11c-positive/CD206-negative M1 macrophages, and F4/80-positive/CD11c-negative/CD206-positive M2 macrophages on days 1, 3, and 7 after priming. *n* = 8; ^∗^*p* < 0.05, ^∗∗^*p* < 0.01, and ^∗∗∗^*p* < 0.001 primed vs. nonprimed SVFs. SVF: stromal vascular fraction.

**Figure 5:**
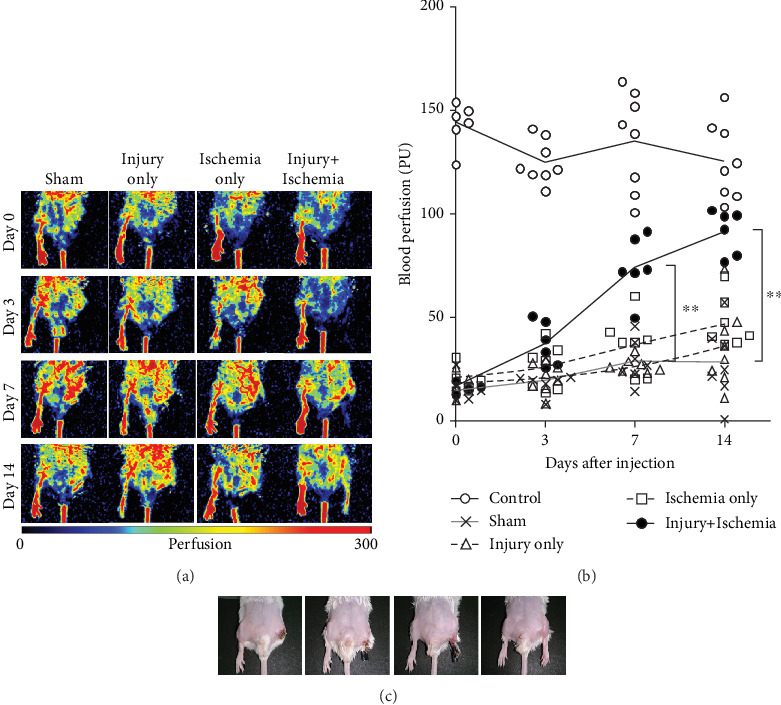
A combination of surgical injury and ischemia was necessary for efficient blood flow recovery during limb ischemia. Blood flow recovery following injection of primed SVFs. (a) Representative laser Doppler blood perfusion imaging. Compared to the sham group (without SVF transplantation), the surgical injury-only group or the ischemia-only group did not show improved blood flow recovery on days 3, 7, and 14 after SVF injection. In contrast, efficient blood flow recovery was achieved in the injury+ischemia group. (b) Quantitative assessment of serial changes in blood perfusion. There was no significant difference in blood perfusion among the sham group, the injury-only group, and the ischemia-only group. The injury+ischemia group showed significantly higher blood perfusion than the sham group, the injury-only group, and the ischemia-only group on days 7 and 14 after injection. (c) Representative appearance of the ischemic (left) and nonischemic (right) hindlimbs. Note that the ischemic limb treated with both injury and ischemia retained an almost intact appearance. ^∗∗^*p* < 0.01. SVF: stromal vascular fraction.

**Figure 6:**
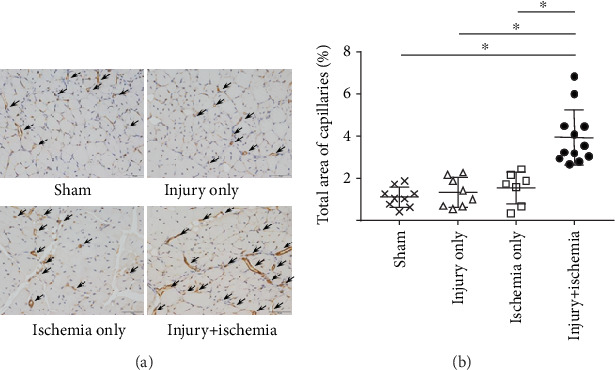
A combination of surgical injury and ischemia was necessary for capillary angiogenesis during limb ischemia. Angiogenesis was visualized by immunohistochemical staining against CD31. (a) Representative microscopic images of CD31-positive capillaries located in ischemic hindlimb muscles. Compared with the sham group (without SVF transplantation), the injury+ischemia group showed increased CD31-positive capillary density on day 14 after injection. The arrows indicate capillaries in the hindlimb muscles. Scale bars = 50 *μ*m. (b) Total area of CD31-positive capillaries per 400x field in sections of the adductor muscle (5 microscopic fields per slide for each mouse); ^∗^*p* < 0.01. SVF: stromal vascular fraction.

**Figure 7:**
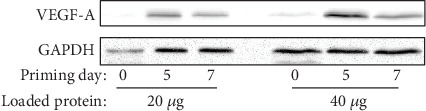
Protein synthesis of VEGF-A was induced by a combination of surgical injury and ischemia in SVFs. 20 *μ*g (left) or 40 *μ*g (right) of whole-cell protein lysate was loaded per lane. Western blotting against VEGF-A or GAPDH was performed. GAPDH served as an internal control. Note that the basal level of VEGF-A synthesis was negligible on day 0 of the priming and the peak of the induction was on 5 days after priming procedure. VEGF: vascular endothelial growth factor; GAPDH: glyceraldehyde 3-phosphate dehydrogenase; SVF: stromal vascular fraction.

To gain biological insight into SVF priming, we performed microarray and pathway analyses. We compared the gene expression profile of primed SVFs with that of nonprimed SVFs (i.e., day 1 or day 3 vs. day 0 after priming). Canonical pathway analysis clearly highlighted innate immunity-related signals such as triggering receptor expressed on myeloid cells (TREM), genes related to the acute phase response, and Toll-like receptor signaling, on both day 1 and day 3 after priming (Figure 2(a)). Consistent with these results, cytokines and receptors for innate immunity were representative upstream regulators during priming (Figure 2(b)). On day 1 after priming, prominent biological alterations included “hematological system development,” “inflammatory response,” “cellular movement,” and “immune cell trafficking” (Figure 2(c)). On day 3 after priming, other biological alterations such as “organismal injury and abnormality” and “cancer” were also prominent (Figure 2(c)). Cell cycle-related signals (e.g., cyclins and cell cycle regulation and mitotic roles of Polo-like kinase) were upregulated exclusively in the later time point (Figure 2(a)). Since canonical pathway analysis suggested the involvement of hypoxia-related signals (e.g., glycolysis pathways), we examined the protein stabilization of hypoxia-induced factor- (HIF-) 1 alpha in the cutaneous fat *in situ*, from which the SVFs have been isolated. On day 1 after the surgical procedure (injury and ischemia), remarkable stabilization of HIF-1 alpha was observed (Figures 3(a) and 3(d)). However, either injury or ischemia alone was not sufficient for the phenomenon (Figures 3(b) and 3(c)). These data indicate that priming induces drastic changes in cellular composition through bone marrow recruitment by innate immunity signals, and subsequently, SVFs optimize their repair function through cell-to-cell interactions and proliferation. Thereafter, we investigated wound repair priming from two different aspects: changes in cellular composition (Figure 4) and functional augmentation in angiogenesis (Figures 5–7).

### 3.3. Priming (Injury+Ischemia) Changed the Heterogeneous Cellular Composition of SVFs

As SVFs consist of a heterogeneous cell population, we evaluated the percentage of CD45-positive mononuclear cells, CD31-CD45-double negative mesenchymal cells [14], mesenchymal-gated CD34-positive adipose progenitors, and F4/80-positive macrophages on days 1, 3, and 7 after priming. The percentage of CD45-positive cells was 92.86 ± 4.09% (range, 85.46–97.42%) on day 1, 90.11 ± 7.67% (range, 78.38–98.78%) on day 3, and 83.85% ± 8.05% (range, 66.75–91.38%) on day 7 (Figure 4(a)). On day 7 after priming, the CD45-positive fraction was significantly lower compared with that of nonprimed SVFs (Figure 4(a)). The percentage of CD31-CD45-double negative cells was 3.40 ± 1.34% (range, 1.85–6.13) on day 1, 3.97 ± 3.07% (range, 0.71–8.61) on day 3, and 9.89 ± 4.09% (range, 5.49–18.83) on day 7 (Figure 4(a)). On day 7 after priming, the CD31-CD45-double negative fraction was significantly higher compared with that of nonprimed SVFs (Figure 4(a)). A scatter plot of two variables (CD45-positive and CD31-CD45-double negative) clearly segregated primed SVFs over nonprimed SVFs (Figure 4(b)).

It is known that the CD31-CD45-double negative fraction include CD34-positive cells with great variability, from 3.5% [15] to 80% [16]. The CD34-positive cells in the CD31-CD45-double negative fraction represent adipose-derived progenitors. The percentage of CD34-positive cells in the CD31-CD45-double negative fraction was 40.07 ± 10.76% (range, 23.02–55.95) on day 1, 22.00 ± 12.06% (range, 7.59–33.33) on day 3, and 45.12 ± 23.23% (range, 10.36–76.73) on day 7. CD34-positive cells were significantly lower on day 3 after priming but were similar on day 7 compared with nonprimed SVFs (Figure 4(c)). Adipose progenitors presenting CD140A (PDGFR alpha) are known to differentiate either into adipocytes or into fibroblasts depending on their physiological context [16]. We measured the CD140A-CD9-double positive cells as the preadipocyte fraction and the CD140A-positive/CD9-negative cells as the prefibroblast fraction. The percentage of CD140A-CD9-double positive cells in the CD31-CD45-double negative fraction was 37.31 ± 10.49% (range, 20.43–55.17) on day 1, 28.47 ± 9.12% (range, 13.25–41.04) on day 3, and 31.88 ± 15.62% (range, 12.81–57.14) on day 7. The percentage of CD140A-positive/CD9-negative cells in the CD31-CD45-double negative fraction was 21.7 ± 11.73% (range, 10.26–48.75) on day 1, 31.56 ± 14.23% (range, 17.1–55.13) on day 3, and 34.22 ± 11.22% (range, 22.86–57.26) on day 7. While the preadipocyte fraction was lower on day 1 after priming, the prefibroblast fraction after priming was similar compared with that of nonprimed SVFs (Figure 4(c)).

The percentage of the F4/80-positive fraction (macrophages) was 22.37 ± 12.71% (range, 5.33–38.46) on day 1, 23.13 ± 17.12% (range, 3.12–44.47) on day 3, and 24.06 ± 9.27% (range, 4.63–34.29) on day 7. The percentage of M1 macrophages (F4/80-positive/CD11c-positive/CD206-negative fraction) was 2.59 ± 1.64% (range, 1.2–6.1) on day 1, 3.39 ± 1.46% (range, 1.15–5.19) on day 3, and 8.62 ± 3.41% (range, 4.67–14.36) on day 7, and that of M2 macrophages (F4/80-positive/CD11c-negative/CD206-positive fraction) was 45.69 ± 20.27% (range, 21.82–77.04) on day 1, 41.34 ± 8.86% (range, 27.48–54.29) on day 3, and 39.03 ± 11.28% (range, 21.83–52.37) on day 7. The percentage of macrophages was significantly higher on days 1 and 7 after priming, whereas the fractions of both the M1 and M2 macrophages were almost similar compared with those of nonprimed SVFs (Figure 4(d)).

### 3.4. A Combination of Surgical Injury and Ischemia Was Necessary for Efficient Blood Flow Recovery and Regeneration of Ischemic Limbs

To quantify the angiogenetic capacity of the primed SVFs, we transplanted SVFs into syngeneic hindlimb ischemia mice. We prepared four different experimental groups to see the essential factors for the priming. After ligation of the right external iliac artery (day 0), ischemic hindlimb blood perfusion was significantly lower than that of the contralateral control hindlimbs in all groups (Figure 5(a)). On days 3, 7, and 14 after SVF injection, the sham group (without SVF transplantation), the injury-only group, and the ischemia-only group did not show an increase in blood flow recovery (Figures 5(a) and 5(b)). In contrast, the injury+ischemia group (74.32 ± 14.82 PU on day 7, 91.51 ± 10.75 PU on day 14) showed significantly higher blood perfusion than the sham group (vs. 28.82 ± 10.41 PU on day 7, vs. 28.40 ± 18.13 PU on day 14; *p* < 0.01), the injury-only group (vs. 26.39 ± 3.56 PU on day 7, vs. 36.08 ± 19.24 PU on day 14; *p* < 0.01), and the ischemia-only group (vs. 33.83 ± 13.75 PU on day 7, vs. 43.27 ± 12.19 PU on day 14; *p* < 0.01) on days 7 and 14 after injection. On day 14, the blood perfusion in the injury+ischemia group was almost equivalent to that of the contralateral control limbs (Figure 5(b)). Consistently, the ischemic limb skin appearance of the four groups on day 14 showed a remarkable improvement. There was no drop-out or scar appearance on the ischemic limb skin in the injury+ischemia group on day 14, while we could see dropping-out of the right ischemic limb in the sham group and scarring in the injury-only group and ischemia-only group (Figure 5(c)).

### 3.5. A Combination of Injury and Ischemia Was Necessary for Capillary Angiogenesis during Limb Ischemia

Finally, we determined the angiogenetic capacity of SVFs by measuring capillary density.  Figure 6(a) shows representative microscopic images for CD31-positive (endothelial cell) capillaries located within ischemic hindlimb muscles on day 14 after SVF injection. The injury+ischemia group (3.94% ± 1.32%) showed a significantly larger area for capillaries than the sham group (vs. 1.11% ± 0.48%; *p* < 0.01), the injury-only group (vs. 1.33% ± 0.70%; *p* < 0.01), and the ischemia-only group (vs. 1.54% ± 0.76%; *p* < 0.01) on day 14 after injection (Figure 6(b)). Vascular endothelial growth factor- (VEGF-) A synthesis was negligible on day 0 of the priming, and the peak of the induction was on 5 days after priming procedure (Figure 7). These results indicate that the combination of injury and ischemia induces capillary angiogenesis through the synthesis of VEGF-A.

## 4. Discussion

Injured tissues release various wound-related factors that affect repair and remodeling [17–19]. Autologous transplantation of adipose-derived SVFs to ischemic limbs promotes angiogenesis and regeneration of ischemic tissues in humans [20] as well as in mouse models [7]. In mouse models, cytokines such as interleukin 6, granulocyte/macrophage colony-stimulating factor, basic fibroblast growth factor, platelet-derived growth factor-bb, vascular endothelial growth factor, and hepatic growth factor are significantly increased in the peripheral blood on day 1 after transplantation of the adipose-derived SVFs [7]. On day 7 after transplantation, however, these cytokines decrease to the same levels as in control mice. This suggests that the transplantation of adipose-derived SVFs improves ischemia via the following two steps: (1) a systemic inflammatory response mobilizes the inflammatory cells from bone marrow, and (2) the transplanted adipose-derived stem/stromal cells and the mobilized inflammatory cells synergistically induce angiogenesis [7].

We incidentally discovered that adipose-derived SVFs in severely injured mice are increased in number and have enhanced angiogenetic capacity through syngeneic transplantation experiments. We subsequently developed a method to reproduce this phenomenon and investigated its mechanisms. The wound repair priming of SVFs requires two surgical procedures: mincing fat parenchyma (injury) and ligating the subcutaneous fat-feeding artery (ischemia). When either one (injury or ischemia) was lacking, the enhancement of angiogenesis in limb ischemia was limited. In the presence of both injury and ischemia, however, we observed enhanced angiogenesis and regeneration of ischemic limbs. After the priming, the cell number of SVFs increased, and microarray analysis showed remarkable changes in the gene expression profile. Thereafter, we performed subsequent experiments to elucidate the molecular and cellular basis of wound repair priming. Microarray data clearly indicated the extensive upregulation of innate immunity signals, suggesting the involvement of the acute inflammatory response. Damage-associated molecular patterning is a possible trigger that induces the inflammatory response [21, 22]. Moreover, hypoxia-induced factor- (HIF-) 1 alpha was stabilized in cutaneous fat *in situ* on day 1 after the priming. The primed SVFs subsequently reorganized heterogeneous cellular compositions and synthesized abundant vascular endothelial growth factor- (VEGF-) A protein through days 5-7. The data support our hypothesis that the transplantation of the primed SVFs improves ischemia in two steps: mobilizing immune cells through a systemic response and synergistic cooperation of heterogeneous cell populations, which contributes to efficient angiogenesis.

In the present study, flow cytometry analysis demonstrated that the cellular composition of primed SVFs changed over time. After priming, the CD45-positive mononuclear cell fraction decreased, but the CD31-CD45-double negative mesenchymal cell fraction increased on day 7 (Figures 3(a) and 3(b)). Recent reports suggest that activated tissue stem cells change the macrophage fraction from M1 to M2 [23]. Since M2 macrophages are known to show tissue remodeling effects [24, 25], this suggests that M2 macrophages could contribute to angiogenesis and regeneration of ischemic limbs. In our results, although the F4/80-positive macrophage fraction increased after priming, the proportion of M1 (F4/80-positive/CD11c-positive/CD206-negative fraction) and M2 (F4/80-positive/CD11c-negative/CD206-positive fraction) cells did not change. Another important stromal component is adipose progenitors, i.e., preadipocytes [26]. Shook et al. recently reported that a subset of myofibroblasts change their number through the proliferation [26] of preadipocytes. However, we observed that the fraction of preadipocytes was decreased transiently, and that of prefibroblasts did not change after priming. We should perform further investigations regarding the specific cellular components of primed SVFs on the regeneration of ischemic limbs.

The processing of human adipose-derived SVFs has been automated for angiogenesis therapy, and autologous transplantation of adipose-derived SVFs is now performed in various clinical settings. However, concerns over individual variations in cell number and quality remain an unsolved issue. Although we have provided a unique concept known as “wound repair priming” and have generated a reproducible surgical procedure, adopting this methodology for humans is unlikely to be feasible. In the future, the priming procedure should be replaced by chemical or pharmacological approaches, the effect of which is equivalent to surgical priming. For example, priming with chemical compounds that stimulate innate immune signals would be a promising alternative. Such chemical or pharmacological priming might contribute to the development of reliable and effective angiogenic therapy for ischemic cardiovascular diseases such as critical limb ischemia.

## 5. Conclusions

Surgical priming with injury+ischemia of adipose tissue results in increased cell numbers and better quality of adipose-derived SVFs. The primed SVFs rapidly reorganize their cell components during wound repair. Residential stromal cells and mobilized immune cells collaborate to achieve effective angiogenesis in ischemic tissues.

## Data Availability

The microarray data obtained in this study have been deposited in the Gene Expression Omnibus (accession number: GSE 134613). Other data used to support the findings of this study are available from the corresponding author upon request.
